# Extracellular ATP Signaling Is Mediated by H_2_O_2_ and Cytosolic Ca^2+^ in the Salt Response of *Populus euphratica* Cells

**DOI:** 10.1371/journal.pone.0053136

**Published:** 2012-12-28

**Authors:** Jian Sun, Xuan Zhang, Shurong Deng, Chunlan Zhang, Meijuan Wang, Mingquan Ding, Rui Zhao, Xin Shen, Xiaoyang Zhou, Cunfu Lu, Shaoliang Chen

**Affiliations:** 1 College of Biological Sciences and Technology, Beijing Forestry University, Beijing, China; 2 College of Life Science, Jiangsu Normal University, Xuzhou, China; Iowa State University, United States of America

## Abstract

Extracellular ATP (eATP) has been implicated in mediating plant growth and antioxidant defense; however, it is largely unknown whether eATP might mediate salinity tolerance. We used confocal microscopy, a non-invasive vibrating ion-selective microelectrode, and quantitative real time PCR analysis to evaluate the physiological significance of eATP in the salt resistance of cell cultures derived from a salt-tolerant woody species, *Populus euphratica*. Application of NaCl (200 mM) shock induced a transient elevation in [eATP]. We investigated the effects of eATP by blocking P2 receptors with suramin and PPADS and applying an ATP trap system of hexokinase-glucose. We found that eATP regulated a wide range of cellular processes required for salt adaptation, including vacuolar Na^+^ compartmentation, Na^+^/H^+^ exchange across the plasma membrane (PM), K^+^ homeostasis, reactive oxygen species regulation, and salt-responsive expression of genes related to K^+^/Na^+^ homeostasis and PM repair. Furthermore, we found that the eATP signaling was mediated by H_2_O_2_ and cytosolic Ca^2+^ released in response to high salt in *P. euphratica* cells. We concluded that salt-induced eATP was sensed by purinoceptors in the PM, and this led to the induction of downstream signals, like H_2_O_2_ and cytosolic Ca^2+^, which are required for the up-regulation of genes linked to K^+^/Na^+^ homeostasis and PM repair. Consequently, the viability of *P. euphratica* cells was maintained during a prolonged period of salt stress.

## Introduction

Plant tolerance to salinity is mediated by a multi-trait, regulatory network. In recent years, plant regulation of ion homeostasis has received much attention. Ca^2+^ and H_2_O_2_ have been widely considered as second messengers involved in salt stress signaling [1]–[5]. Salt treatment generates transient calcium signals to activate salt overly sensitive (SOS) Ca^2+^ sensors that participate in the SOS3-SOS2-SOS1 signaling cascades in Arabidopsis, rice, and poplar [6]–[8]. In the SOS pathway, plasma membrane (PM) Na^+^/H^+^ antiporters (SOS1) play a crucial role in active Na^+^ extrusion under saline conditions [9]–[13]. Ca^2+^ signaling was also shown to be essential for cytosolic Na^+^ detoxification; i.e., the Ca^2+^ sensor, SOS3 complexed with the protein kinase, SOS2, can interact with the Na^+^/H^+^ exchanger, NHX1, and the vacuolar H^+^-ATPase [14], [15]; these ion transporters contribute to vacuolar Na^+^ compartmentation. Recently, H_2_O_2_ has been implicated in the mediation of K^+^/Na^+^ homeostasis in salt-tolerant poplar cells [1], [4]. H_2_O_2_ stabilized *SOS1* mRNA [2] and activated PM Ca^2+^-permeable channels in Arabidopsis [16]. In coordination with Ca^2+^, H_2_O_2_ was suggested to upregulate the activity of the PM H^+^-ATPase, which is fundamental to plant salt tolerance [4]. The H^+^-ATPase was shown to create an H^+^ gradient for Na^+^/H^+^ exchange at the PM; furthermore, a high H^+^-pumping activity inhibited K^+^ efflux through depolarization-activated K^+^ channels in the face of high salinity [6], [17]–[19]. We previously studied callus cells that originated from a salt-sensitive poplar species; those cells lacked the early H_2_O_2_ production typical in response to a salt shock; as a result, K^+^/Na^+^ homeostasis was no longer retained during the following 24-h of salt stress [5].

In plant cells, extracellular ATP (eATP) has been postulated to serve as a signal in growth and stress responses [20], [21]. Previous studies have shown that eATP was involved in the regulation of cotton fiber growth [22], root hair and pollen tube growth [23], [24], stomatal movements [25], [26], auxin transport and root gravitropism [27], membrane potential responses [28], gene expression [29]–[31], and resistance to biotic stress [30], [32]. Furthermore, ATP signaling was shown to be mediated through second messengers, including cytosolic Ca^2+^ ([Ca^2+^]_cyt_), reactive oxygen species (ROS), and NO [31], [33], [34]. Exogenously applied ATP induced an increase in [Ca^2+^]_cyt_ and ROS production in Arabidopsis, and these ATP-mediated responses were blocked with antagonists of animal purinergic receptors (P2 receptors) [31], [33], [35]. These findings suggested that the site of eATP perception may reside at the PM [35], although, to date, no plant purinoceptors have been identified [36]. Exposing plants to NaCl stress was found to produce a significant increase in [eATP] [29], [37]. However, the correlation between eATP and salt resistance has not been established in plants.

In this study, we attempted to clarify the contribution of eATP to salinity tolerance in higher order plants. We used an ideal model system: cell cultures of a salt-resistant woody species, *Populus euphratica*. Callus cells of *P. euphratica* have exhibited high efficiency in regulating K^+^/Na^+^ and ROS homeostasis under salt stress [1], [4], [5], [38]. In this study, we investigated the effects of NaCl on ATP release in the extracellular matrix (ECM), and we aimed to clarify the roles of salt-induced eATP in ion homeostasis and antioxidant defense. Furthermore, because the salt response in higher order plants is typically mediated by H_2_O_2_ and [Ca^2+^]_cyt_ [1]–[5], we determined whether these second messengers contributed to eATP-mediated salinity tolerance. Based on the result from a variety of pharmacological agents, we proposed a speculative model for eATP-mediated salt stress signaling in plant cells.

## Materials and Methods

### Plant Material

Cell cultures of *Populus euphratica* Oliver were prepared as described previously [4], [5]. In brief, callus cells were grown in a Murashige and Skoog (MS) solid medium (2.5% sucrose, pH 5.7), supplemented with 0.25 mg L^−1^ benzyladenine (BA) and 0.50 mg L^−1^ α-naphthaleneacetic acid (NAA), and raised in the dark at 25°C. Callus cells were subcultured every 15 days, and all experiments were performed at 10 days after cells were transferred to fresh propagation medium. Prior to experimental treatments, cell cultures were suspended in liquid MS (LMS) medium without hormones for 1 h equilibration (BA and NAA were removed to reduce potential interactions between the hormones and pharmacological agents applied at the µM range) [4]. Our data showed that the absence of hormones did not significantly change cell viability, H_2_O_2_, and Ca^2+^ flux during 24 h experiment (Fig. S1).

### Treatments

We conducted three series of pharmacological experiments with cells suspended in LMS, as described below. In these pharmacological studies, eATP was depleted with a trap system that comprised 50 mM glucose and 100 units/mL hexokinase (H-G, 6 h); in the H-G trap, hexokinase phosphorylates glucose in a reaction that consuming one molecule of ATP [30]. In no-salt cells, H-G treatment did not markedly change cell viability, H_2_O_2_, Ca^2+^ flux (Fig. S2), activity of antioxidant enzymes (Fig. S3), and expression of salt-responsive genes (Fig. S4). Furthermore, two animal P2 receptor antagonists, suramin and PPADS, were used to block ATP signaling [33]. Concentration tests showed that 10–300 µM of suramin and PPADS had no effect on cell viability or H_2_O_2_ production after 24 h of treatment (Fig. S5). Compared to the low concentrations that we applied (10, 30, 50, 100, and 200 µM), suramin and PPADS at 300 µM exhibited a more pronounced inhibition of the early H_2_O_2_ burst elicited by application of NaCl (200 mM) and non-hydrolyzable ATP (ATP_λ_S, 200 µM; Fig. S6). Therefore, we adopted a working concentration of 300 µM suramin and PPADS, which abolished salt- and ATP-stimulated early H_2_O_2_ in *P. euphratica*, but had no inhibitory effect on cell viability over the observation period.

#### Series 1: Long-term pharmacological experiments (24h)

Suspended cells were pretreated without or with suramin (300 µM for 2 h), PPADS (300 μΜ for 2 h), or an H-G system (50 mM glucose and 100 units/mL hexokinase for 6 h), followed by the addition of NaCl (200 mM). After 24 h, we measured cell viability, H_2_O_2_ accumulation, membrane potential (MP), Na^+^ levels in the cytosol and vacuole, expression levels of salt-responsive genes, and steady-state fluxes of Na^+^, H^+^, and K^+^. Activities of antioxidant enzymes (catalase, CAT; ascorbic peroxidase, APX; glutathione reductase, GR) were examined in untreated control and saline-stressed cells that had been pretreated with or without suramin, PPADS, or H-G.

#### Series 2: Short-term pharmacological experiments

Suspended cells were subjected to suramin or PPADS (300 µM) for 2 h or the H-G solution (50 mM glucose and 100 units/mL hexokinase) for 6 h prior to the addition of NaCl (200 mM). Immediately after NaCl addition, during the following observation period (30–60 min), we recorded H_2_O_2_, cytosolic Ca^2+^, Na^+^ compartmentation, and transient fluxes of H^+^, K^+^, and Ca^2+^ across the PM.

#### Series 3: Pharmacological experiments with exogenous ATP application

In this series, ATP was introduced to inhibitor-pretreated cells to confirm that eATP mediated the salt response in *P. euphratica* cells. The pharmacological experiments were designed as described in Series 1 and 2, except that different concentrations of ATP (10, 50, 100, and 200 µM) were added to the 200 mM NaCl solution. The addition of 200 µM ATP exhibited a pronounced rescue from the H-G inhibition; both early H_2_O_2_ production (30 min) and late Na^+^ extrusion (24 h) were rescued in the presence of high salinity (Fig. S7). Therefore, we adopted a working concentration of 200 µM ATP. During the short-term (30–60 min) salt exposure in Series 2, we measured H_2_O_2_, cytosolic Ca^2+^, and transient fluxes of H^+^, K^+^ and Ca^2+^. After the long-term (24 h) treatment of Series 1, we measured cell viability, H_2_O_2_ accumulation, MP, Na^+^ levels in the cytosol and vacuole, expression of salt-responsive genes, and steady-state fluxes of Na^+^, H^+^, and K^+^.

### ATP Release Assays

ATP levels in the ECM were measured with the Enlighten ATP assay system bioluminescence kit (Promega, Madison, WI, USA). In brief, *P. euphratica* cells (0.1 g) were suspended in 0.5 mL LMS that contained a P2 receptor antagonist (suramin or PPADS, 300 µM) or the H-G solution (LMS supplemented with 50 mM glucose and 100 units/mL hexokinase). After incubation at room temperature for 2 h or 6 h, respectively, the medium was exchanged with a solution of high NaCl (200 mM) prepared in LMS with the corresponding inhibitors. Control cells were not exposed to NaCl or pharmacological agents. Cell-free supernatants were collected at the indicated time points and immediately frozen in liquid nitrogen for later analyses. ATP was determined in an assay with luciferin-luciferase. All samples were assessed with a Turner Designs Modulus™ Microplate Multimode Reader (Promega Corp., Madison, WI, USA). Two individual 10 µL samples were assayed from each replicate to ensure internal consistency of the sample. The concentration of eATP was calculated from a standard curve; the [eATP] varied over a linear range of 0.01 to 100 nM.

### Assessment of Cell Viability

Cell viability was measured with a fluorescein diacetate stain (FDA; Sigma-Aldrich), as described previously [5]. Cell suspensions from Series 1 and 3 were stained with 20 µg mL^−1^ FDA (Sigma-Aldrich) and then incubated in the dark for 10 min at room temperature. Samples were observed under a Leica inverted fluorescence microscope (Leica Microsystems GmbH, Wetzlar, Germany) at an excitation wavelength of 480 nm. Cell viability was calculated by counting 8–10 randomly selected fields, each, with at least 300 cells.

### Detection of H_2_O_2_

The specific fluorescence of H_2_O_2_ was detected with dichlorodihydrofluorescein diacetate (H_2_DCF-DA; Molecular Probes, Eugene, OR) [4], [5]. Suspended cells pretreated with or without pharmacological agents (suramin, PPADS, H-G, glucose) were treated with NaCl (200 mM) or NaCl (200 mM)+ATP (200 µM) for 24 h (Series 1 and 3). Then, cells were fixed on poly-L-lysine-pretreated cover slips (2×5 cm) and treated with 50 µM H_2_DCF-DA (prepared in LMS) for 5 min at room temperature in the dark. Then the H_2_DCF-DA-loaded cells were washed 3–4 times with LMS and analyzed with a Leica SP5 confocal microscope (Leica Microsystems GmbH, Wetzlar, Germany). The confocal settings were as follows: excitation 488 nm, emission 510–530 nm, frame 512×512. Three-dimensional (3D) scanning was performed with a 3-µm Z-series project step, and 3D reconstructed images of cells were used to calculate relative fluorescence. Image processing software (Adobe Systems; Leica Application Suite Advanced Fluorescence; Leica Microsystems) was used to determine the fluorescent intensity of all the individual cells, and each measurement was expressed as the number of pixels on a scale of 0 to 255.

We also recorded the transient response of H_2_O_2_ to NaCl. Control or inhibitor-pretreated cells from Series 2 and 3 were loaded with 50 µM H_2_DCFDA for 5 min prior to the addition of NaCl, supplemented with or without ATP. DCF-dependent fluorescence was measured every 5 min with a confocal microscope.

### Detection of Membrane Potential (MP)

The MP was detected with a fluorescent probe, Bis-(1,3-dibutylbarbituric acid)trimethine oxonol (DiBAC_4_(3); Molecular Probe, Eugene, OR, USA) [4], [39]. A stock solution of DiBAC_4_(3) (200 µM in DMSO) was added to suspended cells that had been treated with NaCl, inhibitors, and ATP (Series 1 and Series 3); the final concentration of DiBAC_4_(3) was 2 µM (10 min). A total of 200 µL cells were placed in the centers of poly-L-lysine-pretreated cover slips (2×5 cm), and DiBAC_4_(3)-dependent fluorescence was measured with a confocal microscope. The confocal settings were the same as those described above for H_2_O_2_ detection [4], [5].

### Visualization of Intracellular Na^+^ Levels

To evaluate the effects of eATP on the pattern of intracellular Na^+^ distribution, we used a Na^+^-specific fluorescent dye, CoroNa-Green AM, to visualize Na^+^ within cells [40]. After the treatments were applied in Series 1 and 3, suspended cells were loaded with CoroNa-Green AM (20 µM) for 2 h and analyzed with confocal microscopy. The confocal settings were as follows: excitation 488 nm, emission 510–530 nm, frame 512×512. The Na^+^-specific fluorescence in the cytosolic and vacuolar compartments were calculated with Image-Pro Plus 6.0 software (Media Cybernetics, Bethesda, USA). In addition to the effects of long-term salt stress (24 h), we also examined the effects of suramin, PPADS, and H-G on Na^+^ compartmentation after a short-term treatment (1 h, Series 2).

### Flux Measurements of Na^+^, H^+^, K^+^ and Ca^2+^

Net fluxes of Na^+^, H^+^, K^+^, and Ca^2+^ were measured non-invasively with the Scanning Ion-selective Electrode Technique (the SIET system, BIO-001A, Younger USA Sci. & Tech. Corp., Amherst, MA, USA; Applicable Electronics Inc., Forestdale, MA, USA and ScienceWares Inc., East Falmouth, MA, USA). Recordings of transient H^+^, Ca^2+^, K^+^, and steady-state Na^+^, H^+^, K^+^ fluxes were performed as described previously [4], [12]. For transient H^+^, K^+^, and Ca^2+^ recordings, control or inhibitor-pretreated cells (Series 2 and Series 3) were settled on the bottom of a poly-L-lysine-pretreated petri dish in 4 mL measuring solution (0.5 mM KCl, 0.2 mM CaCl_2_, 0.1 mM MgCl_2_, 0.1 mM NaCl, 2.5% sucrose, pH 5.7), with added H-G, PPADS, and suramin. First, the steady-state H^+^, K^+^, and Ca^2+^ fluxes were recorded (5–6 min) prior to the NaCl and ATP treatment. Stock solutions of NaCl (400 mM) and ATP (400 µM) were slowly added to the measuring solution until the final concentration in the solution reached 200 mM NaCl, with or without 200 µM ATP. The flux recording was restarted and continued for 30–35 min. The data measured during the first 30 s were discarded, due to the diffusion effects of the stock addition (in this study, blank measurements without cells were carried out to identify the time interval in which the addition of stock disturbed the flux measurements). We compared the kinetics of Ca^2+^ transients elicited by 100 and 200 mM NaCl.

For steady-state Na^+^, H^+^, and K^+^ flux measurements, cells pretreated with NaCl and inhibitors (Series 1 and Series 3) were placed in the centers of poly-L-lysine-pretreated coverslips in 4 mL measuring solution (0.5 mM KCl, 0.2 mM CaCl_2_, 0.1 mM MgCl_2_, 200 mM NaCl, 2.5% sucrose, pH 5.7). The steady-state flux measurements were, as a rule, continuously recorded for 8–10 min (Na^+^ flux was recorded in a measuring solution with 0.1 mM Na^+^, as a high Na^+^ concentration in the measuring solution lowered signal/noise ratio of Na^+^ electrodes [12]). For control cells treated without NaCl or inhibitors, fluxes were recorded in the measuring solution (0.5 mM KCl, 0.2 mM CaCl_2_, 0.1 mM MgCl_2_, 0.1 mM NaCl, 2.5% sucrose, pH 5.7). Three-dimensional ionic flux signals were plotted with MageFlux software, developed by Yue Xu (http://xuyue.net/mageflux).

### Detection of Cytosolic Ca^2+^ Levels

Cytosolic Ca^2+^ was visualized with a Ca^2+^-sensitive fluorescent dye, Rhod-2/AM (Biotium) [41]. In brief, suspended cells pretreated with or without pharmacological agents (suramin, PPADS, or the H-G system; Series 2 and 3) were loaded with 2 µM Rhod-2/AM in LMS at 4°C for 60 min. Then, Rhod-2-loaded cells were washed twice with LMS, followed by a 60-min incubation at 25°C with the corresponding inhibitors (suramin, PPADS, or H-G). Next, the cells were subjected to NaCl and ATP treatments, and Rhod-2-specific fluorescence was measured every 30 s with a xyt project step over a period of 30 min (excitation, 543 nm; emission, 570–590 nm). The intensity of the measured compartment (cytoplasm) was calculated with image-processing software (Adobe Systems; Leica Application Suite Advanced Fluorescence; Leica Microsystems).

### Antioxidant Enzyme Extraction and Activity Measurements

We also examined activities of antioxidant enzymes, CAT, APX, and GR, under salt and ATP treatments. *P. euphratica* cells were treated with 200 mM NaCl for 24 h in the absence or presence of corresponding agents (suramin, PPADS, or H-G; Series 1). Control cells were incubated in LMS without addition of NaCl, inhibitors, or ATP (the effect of ATP addition, 200 and 500 µM, on antioxidant enzymes was examined in control cells, Fig. S3). Then, callus samples (0.2 g) were ground to a fine powder in liquid N_2_ and homogenized in 2 mL of 50 mM potassium phosphate buffer (pH 7.0) containing 1 mM EDTA and 1% polyvinylpyrrolidone (PVP) [42]. The homogenate was centrifuged at 10,000×*g* for 20 min at 4°C, and the supernatant was examined for total CAT and GR activities, as described previously [5], [43]. For the APX measurement, 1 mM ascorbic acid (ASA) was added to the enzyme extraction buffer [5], [43]. Protein concentrations were determined as described previously [44], with bovine serum albumin as the standard.

### Quantitative Real-time PCR

Total RNA was isolated with Trizol reagent and purified with a RNA purification kit (Qiagen, RNeasy spincolumn), followed by an on-column DNase treatment. Then, 2 µg of total RNA was reverse transcribed with SuperScript III (Invitrogen) and oligo (dT) primers (Invitrogen). Next, 1 µL of synthesized cDNA was used as template for real-time PCR (RT-PCR) amplification. The PCR products were sequenced and validated for quantitative RT-PCR (qRT-PCR). Primers designed to target *NHX1*, *SOS1*, and *vacuolar H^+^-ATPase-subunit a* (*VHA-a*) were based on expressed sequence tags (ESTs) from *P. euphratica*; and primers designed to target the *PM H^+^-ATPase* (*AHA*), *vacuolar H^+^-pyrophosphatase* (*AVP*), *VHA-b*, *VHA-c*, *mitogen-activated protein kinase* (*MPK*), and *synaptotagmin* (*SYT*) were based on *P. trichocarpa* homologs (Table S1). All the ESTs were obtained from the NCBI database (http://www.ncbi.nlm.nih.gov/guide/). The resulting amplicons were between 150 and 300 bp. The total 25 µL qRT-PCR reaction volume contained 12.5 µL SYBR Green PCR Master Mix (Applied Biosystems), 1 µL of 1∶1 (v/v) diluted cDNA, and 0.12 µM gene-specific primers. The PCR was performed on an Applied Biosystems 7500 Fast Real-Time PCR System (Life Technologies Corp., Carlsbad, CA, USA). The melting-curves were analyzed immediately to confirm the specificity of the products; the mean Ct value for each gene was obtained from three independent PCR experiments. The relative expression level for each target gene was normalized to the *Populus* housekeeping gene, *Actin* (GeneBank number XM_002322628), with the forward primer 5′-CCCTCTATGCCAGTGGTCGTA-3′ and the reverse primer 5′-ACGCTCTGCTGTGGTTGTGAA-3′. Relative expression levels were calculated with the 2^−ΔΔC^ method. In addition to the gene expression elicited by high salt exposure, we examined the effects of suramin, PPADS, H-G, ATP (100 and 200 µM), and ATP_λ_S (200 µM) on the expression of salt-responsive genes in control cells that were not exposed to high salt conditions (Fig. S4).

### Data Analysis

All mean data were subjected to an analysis of variance. Significant differences between means were determined with Duncan’s multiple range test. Unless otherwise stated, differences were considered statistically significant when *p*<0.05.

## Results

### NaCl-induced eATP Mediated Ion Homeostasis and Antioxidant Defense in *P. euphratica* Cells

#### ATP concentration in the ECM

**Figure 1:**
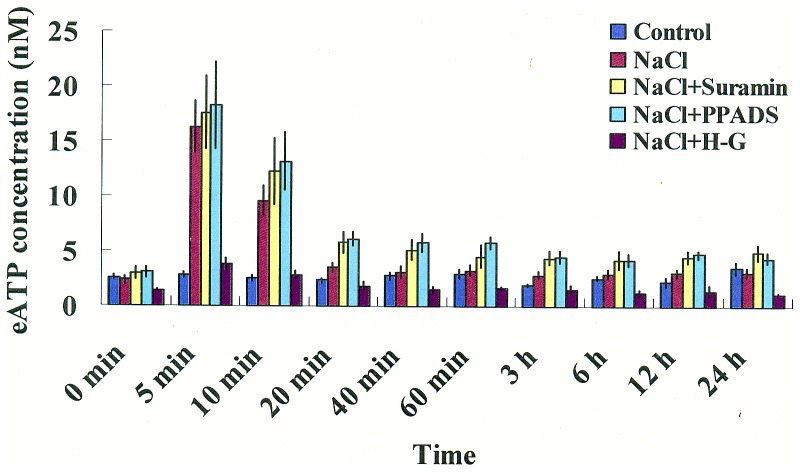
Extracellular ATP levels in *P. euphratica* cells under NaCl stress. Time courses of ATP release in response to high NaCl (200 mM), in the presence or absence of P2 receptor antagonists (suramin or PPADS, 300 μΜ) or an ATP trap (H-G system, 50 mM glucose and 100 units/mL hexokinase). Bars represent the means from five independent experiments and whiskers represent the error of the mean.

It was previously shown in Arabidopsis seedlings that plants responded to increased hypertonic stress with a transient increase in [eATP] [29], [37]. However, the contribution of elevated eATP to salt adaptations has remained unclear. We investigated the role of eATP in salt adaptation of *P. euphratica* by adding suramin or PPADS to block eATP signaling [33] or by depleting eATP with the H-G system [30]. In the present study, we used luciferin-luciferase as a bioluminescent reporter to evaluate eATP levels in the ECM of *P. euphratica* cells. Figure 1 shows that ATP concentrations increased with high NaCl exposure (200 mM). The peak [eATP] occurred at 5 min (16.3±2.4 nM); 5.6-fold over control peaks, then [eATP] returned to control levels at 20 min and remained constant for the remainder of the study (Fig. 1). This transient eATP response to NaCl stress was reduced by the H-G system (Fig. 1). In contrast, application of suramin or PPADS had no effect on the salt-elicited transient increase in [eATP], but they slowed the recovery of [eATP] to basal levels (Fig. 1).

#### Cell viability

**Figure 2:**
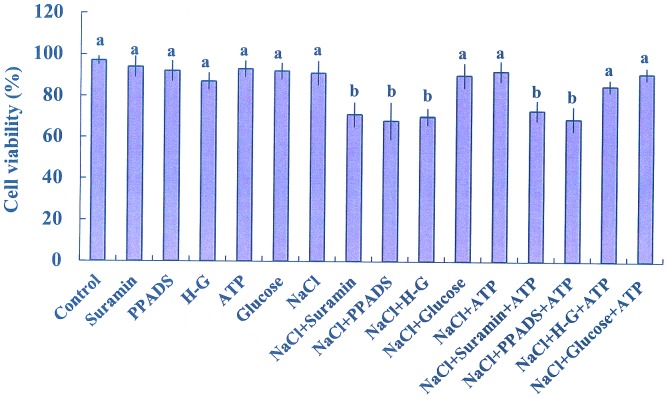
Effects of pharmacological agents, glucose, and ATP on NaCl stress-related viability. Suspended cells, incubated with or without pharmacological agents (suramin, 300 µM; PPADS, 300 µM; and H-G, 50 mM glucose and 100 units/mL hexokinase) or glucose (50 mM), were exposed to NaCl (200 mM) or NaCl plus ATP (200 µM) for 24 h. Control cells were cultured with no addition of NaCl or any pharmacological agent. Bars represent the means of three independent experiments (in each at least 300 cells were counted). Whiskers represent the error of the mean. Different letters (a, b) denote significant differences between treatments (*P*<0.01).

Cell viability was used as an indicator of salinity tolerance in plant cells [5]. To determine whether eATP mediated cell viability under NaCl stress, we examined the effects of P2 receptor antagonists and the ATP trap on the viability of *P. euphratica* cells exposed to high salt. First, we found that a 24-h exposure to NaCl (200 mM) did not suppress cell viability (Fig. 2). However, treatment with suramin, PPADS, or H-G resulted in a significant reduction in viability with 24 h of salt stress (Fig. 2). Similar to H-G and glucose, treatment with suramin or PPADS at concentrations of 10 to 300 µM had no obvious effect on cell viability under control conditions (Figs. 2, S5A). Under salt stress, addition of ATP (200 µM) rescued the H-G-triggered death, but did not alter the effects of suramin or PPADS (Fig. 2). Moreover, the addition of 200 µM ATP did not cause cell death in control cells (Fig. 2), similar to our previous findings [41]. Glucose had no effects on cell viability under NaCl stress, irrespective of ATP treatment (Fig. 2).

#### Na^+^ compartmentation within cells and PM Na^+^/H^+^ antiport

**Figure 3:**
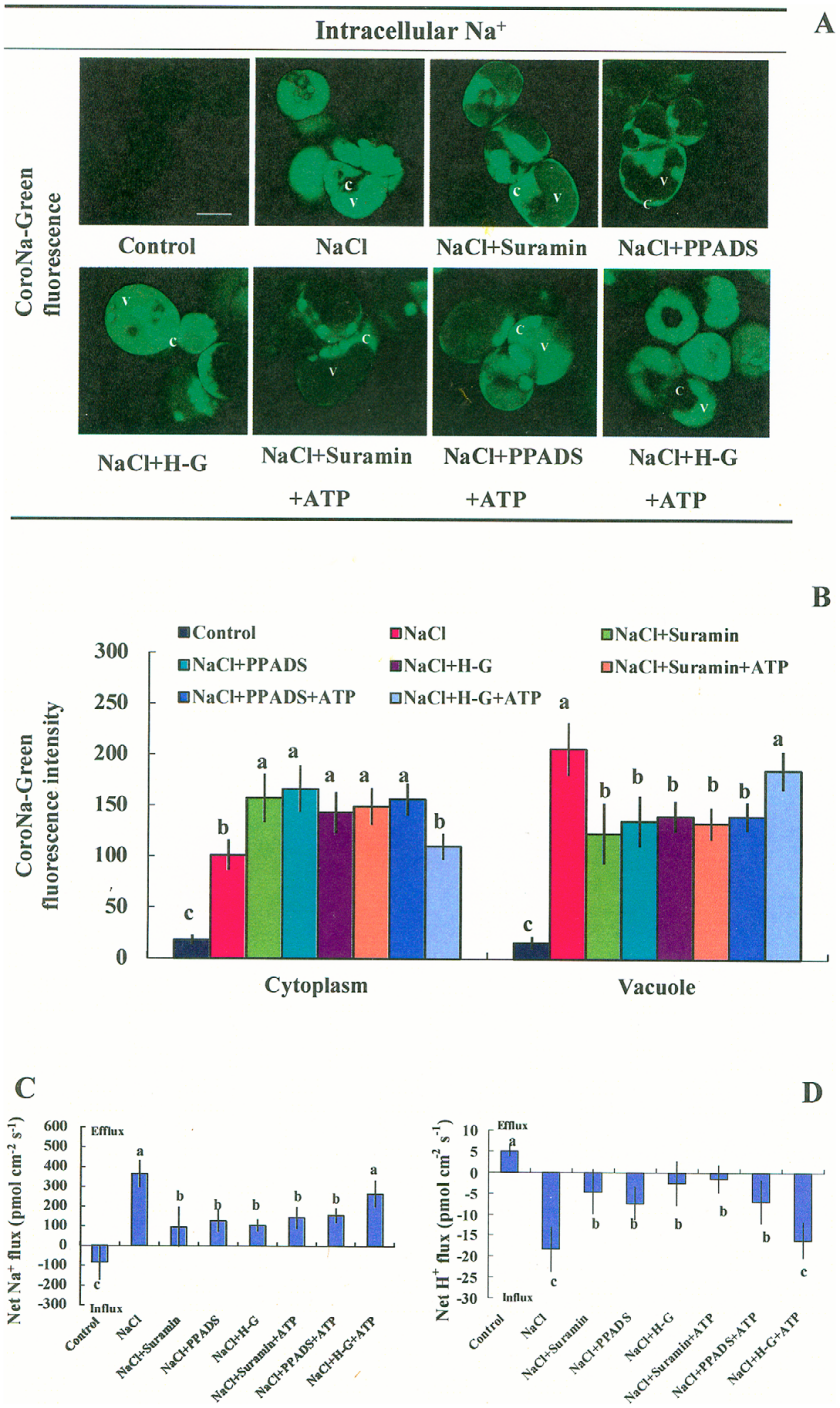
Effects of P2 receptors antagonists on NaCl-induced Na^+^ compartmentation and PM Na^+^/H^+^ antiport. *P. euphratica* cells were untreated (control) or treated with 200 mM NaCl with or without 200 µM ATP for 24 h in the presence or absence of suramin (300 µM), PPADS (300 µM), or the H-G system (50 mM glucose and 100 units/mL hexokinase). Then, cells were stained with the Na^+^-specific fluorescent probe, CoroNa-Green/AM, to detect cytosolic and vacuolar Na^+^ levels. Steady-state Na^+^ and H^+^ fluxes were measured with SIET. (A, B) Na^+^ levels within the cytoplasm (c) and vacuole (v). Bars represent the means of at least 100 individual cells quantified from three independent experiments. (C, D) Steady-state fluxes of Na^+^ and H^+^. Bars represent the mean of 11–13 individual cells from three independent experiments. (B-D) Whiskers represent the standard error of the mean. Different letters (a, b, c) denote significant differences between treatments (*P*<0.05).

Maintenance of Na^+^/K^+^ homeostasis is a remarkable feature of the salt tolerance of *P. euphratica* cells [5]. We explored whether NaCl-induced eATP contributed to Na^+^/K^+^ homeostasis in *P. euphratica* cells. We used a Na^+^-specific fluorescent probe, CoroNa-Green AM, to indicate Na^+^ levels within intracellular compartments. Figure 3A shows that *P. euphratica* cells exhibited a marked increase in CoroNa-Green-specific fluorescence after 24 h of NaCl stress, but Na^+^-specific fluorescence was nearly undetectable in control cells. Of note, more of the Na^+^-specific fluorescence was distributed in the vacuolar region than in the cytoplasm (Fig. 3A,B). However, the pattern of Na^+^ partitioning within cells was altered by the application of suramin, PPADS, or H–G. These agents dramatically reduced the fraction of Na^+^ partitioned to the vacuoles and this paralleled increases in the fraction of Na^+^ partitioned to the cytoplasm (Fig. 3A, B). With ATP (200 µM) supplementation, the NaCl stress-induced pattern of Na^+^ partitioning was rescued in H-G-treated cells, but not in suramin- or PPADS-treated cells (Fig. 3A, B).

After 24 h of NaCl stress, *P. euphratica* cells exhibited marked increases in Na^+^ efflux and H^+^ influx (Fig. 3C, D). This indicated active Na^+^/H^+^ antiport across the PM [4]. However, the NaCl-enhanced PM Na^+^/H^+^ antiport activity was depressed by suramin, PPADS, or H-G (Fig. 3C, D). Interestingly, ATP application (200 µM) rescued the Na^+^ efflux and H^+^ influx in salinized cells that were pretreated with H-G (Fig. 3C,D). In contrast, exogenously applied ATP at concentrations of 10, 50, 100, or 200 µM did not rescue salinized cells pretreated with suramin or PPADS (Figs. 3C, S7A).

Unexpectedly, with short-term salt treatment (1 h), the pattern of Na^+^ compartmentation within the vacuoles and cytoplasm was not altered by the application of suramin, PPADS, or H-G (Fig. S8). This data implied that the regulation of salt-induced eATP on Na^+^ transport across plasma and vacuolar membranes was more pronounced in prolonged NaCl stress as compared to short-term stress.

#### Membrane potential and K^+^ flux

**Figure 4:**
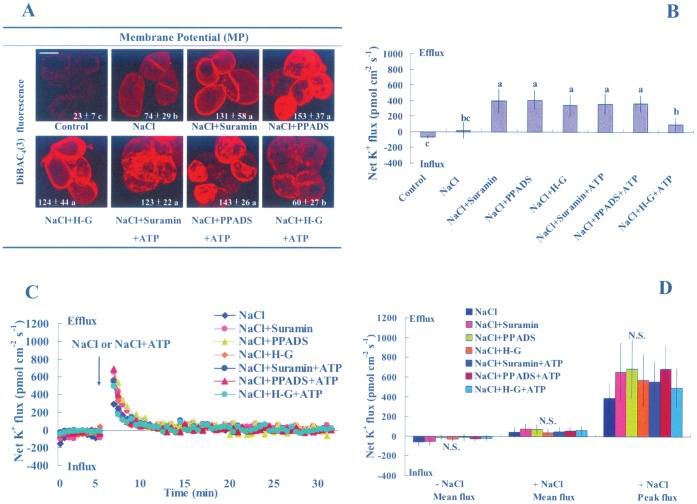
Effects of pharmacological agents on NaCl stress-related membrane potential and steady-state and transient K^+^ fluxes. (A) Membrane potential (MP). *P. euphratica* cells were untreated (control) or treated with 200 mM NaCl supplemented with or without 200 µM ATP for 24 h in the presence and absence of suramin (300 µM), PPADS (300 µM), or the H-G system (50 mM glucose and 100 units/mL hexokinase). Then, cells were incubated with the MP-sensitive fluorescent probe, DiBAC_4_(3). Values (white font) represent the mean±SD based on quantifications from at least 50–60 individual cells in three independent experiments. Different letters (a, b, c) denote significant differences between treatments (*P*<0.01). (B) Steady-state K^+^ fluxes. K^+^ fluxes across the PM were measured with SIET. Bars represent the mean of 15–18 individual cells and whiskers represent the standard error of the mean. Different letters (a, b, c) denote significant differences between treatments (*P*<0.05). (C) Transient K^+^ fluxes in response to NaCl (200 mM) or NaCl (200 mM) plus ATP (200 µM) in the presence and absence of suramin, PPADS, or H-G system. Each point represents the mean of six individual cells measured in three independent experiments. (D) Peak and mean values for transient K^+^ fluxes before (-) and after (+) the addition of NaCl or NaCl plus ATP. Bars represent the mean of six individual cells and whiskers represent the standard error of the mean. N.S. = no significant difference.

K^+^ flux depends on MP in salt-stressed *P. euphratica* cells [4]. In the present study, we investigated whether eATP regulated the MP and K^+^ homeostasis of *P. euphratica* cells exposed to long- and short-term salt stress. After 24 h of high NaCl treatment, the PM was depolarized and K^+^ efflux was increased in *P. euphratica* cells (Fig. 4A,B). This result was consistent with our previous findings [4]. Of note, the salt-induced membrane depolarization and K^+^ efflux were both enhanced by suramin, PPADS, and the H-G system (Fig. 4A,B); this suggested that eATP was involved in mediating K^+^ transport under high saline conditions. Interestingly, ATP supplementation (200 µM) reduced the enhancement in K^+^ efflux and PM depolarization mediated by H-G in salinized cells, but not that mediated by suramin or PPADS treatment (Fig. 4A,B). In contrast, the transient K^+^ efflux elicited by NaCl-shock in *P. euphratica* cells (short-term salt stress) was not affected by suramin, PPADS, H-G, or exogenously applied ATP (Fig. 4C, D).

#### Expression of salt-responsive genes

**Figure 5:**
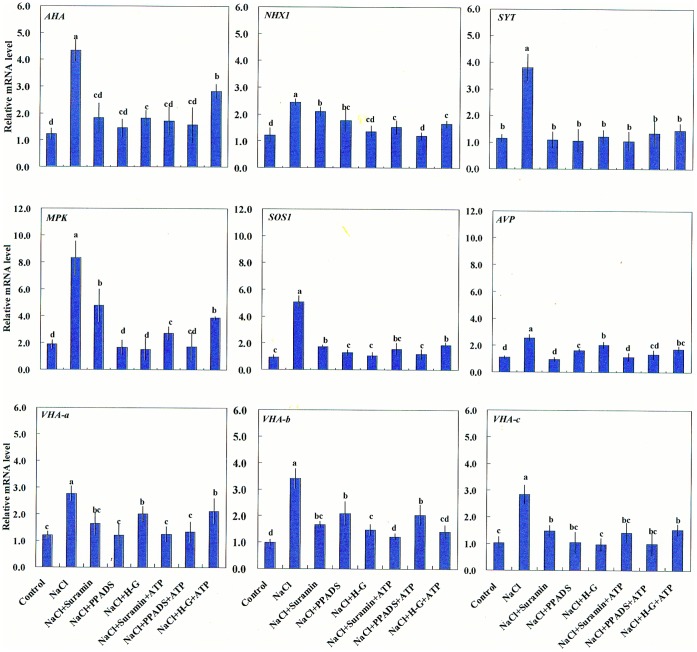
Effects of pharmacological agents on expression of salt-responsive genes in NaCl-stressed *P. euphratica* cells. *P. euphratica* cells were untreated (control) or treated with 200 mM NaCl or NaCl plus 200 µM ATP for 24 h in the absence or presence of suramin (300 µM), PPADS (300 µM), and the H-G system (50 mM glucose and 100 units/mL hexokinase); then, total RNA was isolated for quantitative Real-Time PCR analysis. Bars represent the mean of four replicates and whiskers represent the standard error of the mean. Different letters (a, b, c, d) denote significant differences between treatments (*P*<0.05).

Interestingly, the regulation of salt-induced eATP on Na^+^/K^+^ homeostasis was more pronounced in response to prolonged NaCl stress (24 h) compared to short-term NaCl stress (within 1 h; Figs. 3, 4, S8). These results suggested that salt-induced eATP may regulate gene expression under prolonged high NaCl exposure. Thus we examined salt-induced expression of genes related to the Na^+^/H^+^ antiport system and PM repair. Figure 5 shows that NaCl stress (24 h) induced significant increases in the mRNA expression of nine selected genes, including genes that encode the PM H^+^-ATPase (*AHA*), the PM Na^+^/H^+^ antiporter (*SOS1*), synaptotagmin (*SYT*), mitogen-activated protein kinase (*MPK*), vacuolar Na^+^/H^+^ antiporter (*NHX1*), vacuolar H^+^-pyrophosphatase (*AVP*), and vacuolar H^+^-ATPase subunits a–c (*VHA-a*, *VHA-b* and *VHA-c*). We used suramin, PPADS, and the H-G system to study eATP regulation of the transcription of these salt-responsive genes. We found that, in salinized cells, application of suramin, PPADS, and H-G markedly inhibited the abundance of the mRNA levels of these selected genes (Fig. 5). Furthermore, exogenously applied ATP rescued the H-G-mediated inhibition of *AHA*, *MPK*, *SOS1*, and *VHA-c* transcription in salinized cells, but not the effects of suramin or PPADS (Fig. 5). In this study, suramin, PPADS, and H-G had no obvious effects on gene expression when added in the absence of NaCl stress (Fig. S4). Among the selected genes, only *MPK* was upregulated by ATP (200 µM) or ATP_λ_S (200 µM) under control conditions (Fig. S4).

#### H_2_O_2_ accumulation and activity of antioxidant enzymes

**Figure 6:**
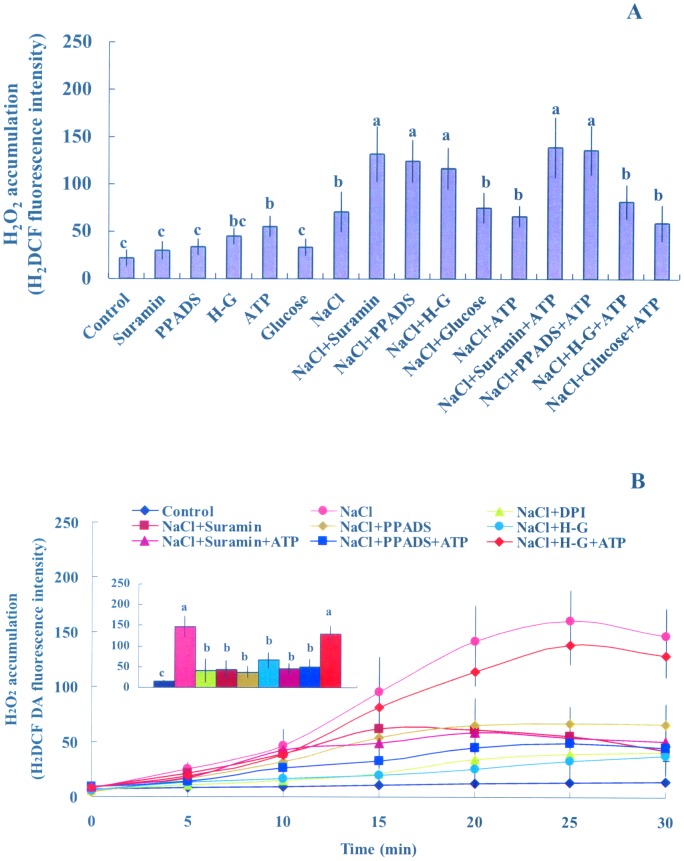
Effects of pharmacological agents and ATP on H_2_O_2_ production in *P. euphratica* cells under NaCl stress. (A) H_2_O_2_ accumulation after 24 h of salt stress. Suspended cells, incubated with or without pharmacological agents (suramin, 300 µM; PPADS, 300 µM; and H-G, 50 mM glucose and 100 units/mL hexokinase) or glucose (50 mM), were exposed to NaCl (200 mM) or NaCl plus ATP (200 µM) for 24 h. Control cells were cultured with no addition of NaCl or any pharmacological agent. Bars represent the means of three independent experiments (in each 45 to 50 individual cells were quantified). Whiskers represent the error of the mean. Different letters (a, b, c) denote significant differences between treatments (*P*<0.01). (B) Early H_2_O_2_ production upon salt shock. Suspended cells were untreated (control) or pretreated without or with DPI (100 µM for 30 min), suramin (300 µM for 2 h), PPADS (300 µM for 2 h), or the H-G system (50 mM glucose and 100 units/mL hexokinase for 6 h), followed by exposure to NaCl (200 mM) with or without ATP (200 µM) supplementation. Transient production of H_2_O_2_ was recorded under a confocal microscope. Each point represents the mean of 15 to 18 individual cells from four independent experiments. Inserted panels show the H_2_DCF-dependent fluorescence intensity after 20–25 min of treatment. Different letters (a, b, c) denote significant differences between treatments (*P*<0.01).

The capacity to maintain ROS homeostasis is crucial for salt adaptation in *P. euphratica* plants [43], [45]. In this study, we examined H_2_O_2_ accumulation in *P. euphratica* cells after 24 h of NaCl stress. DCF-dependent fluorescence indicated that H_2_O_2_ was significantly increased after 24 h of high NaCl treatment (Fig. 6A). Of note, this effect of NaCl on H_2_O_2_ accumulation was enhanced in the presence of suramin, PPADS, or H-G (Fig. 6A). In control cells, H_2_O_2_ was not increased in the presence of H-G or P2 receptor antagonists (10 to 300 µM, Figs. 6A, S5B). ATP (200 µM) increased H_2_O_2_ accumulation in control cells, but no enhancement was detected with ATP in salinized cells (Fig. 6A). Under NaCl stress, the addition of ATP significantly decreased the H_2_O_2_ accumulation elicited by H-G, but not that elicited by suramin or PPADS treatment (Fig. 6A). Glucose had no effect on H_2_O_2_ production under control or saline conditions (Fig. 6A).

In addition, APX, CAT, and GR were up-regulated by 24-h salt stress (Fig. S3). However, addition of suramin, PPADS, or H-G significantly reduced the salt-induced upregulation of enzyme activities (Fig. S3). As a result, the down-regulation of the activity of antioxidant enzymes led to a H_2_O_2_ burst after 24-h of NaCl treatment (Figs. 6A, S3). Addition of ATP at 200 and 500 µM for 24 h also markedly enhanced APX, CAT, and GR activities in the absence of salt stress (Fig. S3). These data indicated that eATP signaling was implicated in antioxidant defense and redox homeostasis in salinized *P. euphratica* cells. Moreover, increased activities of APX, CAT, and GR in salinized *P. euphratica* cells may be a result of an early H_2_O_2_ production (see below, Fig. 6B), because ROS are considered secondary messengers that induce antioxidant defenses [43], [45]–[47].

### Salt-induced eATP Triggers an Early H_2_O_2_ Production and Establishment of Cytosolic Ca^2+^

#### H_2_O_2_ production

Our previous studies revealed that high salt could elicit rapid H_2_O_2_ and cytosolic Ca^2+^ signaling, which contributed to Na^+^/K^+^ homeostasis in *P. euphratica* cells [4], [5]. Furthermore, previous studies at both tissue and cellular levels showed that eATP induced ROS and [Ca^2+^]_cyt_ [31], [33], [35]. Therefore, we reasoned that eATP signaling might be mediated by H_2_O_2_ and cytosolic Ca^2+^ in salinized *P. euphratica* cells. We found that salt stress induced a rapid increase in H_2_O_2_, as indicated by DCF-fluorescence (Fig. 6B). However, this rapid H_2_O_2_ induced by NaCl was markedly reduced by DPI, an inhibitor of PM NADPH oxidase (Fig. 6B). Application of H-G or either of the P2 receptor antagonists showed a trend that was similar to the reduction observed with DPI (Fig. 6B). The inhibition of suramin and PPADS on H_2_O_2_ production depended on the concentration applied (10, 30, 50, 100, 200, or 300 µM; Fig. S6A). The two P2 receptor antagonists also caused a dose-dependent reduction in H_2_O_2_ production elicited by addition of 200 µM ATP_λ_S in the absence of salt stress (Fig. S6B). We also applied exogenous ATP to inhibitor-pretreated cells to confirm the eATP effect on H_2_O_2_ elicited by NaCl stress. Our data showed that addition of ATP (200 µM) rescued the salt-induced H_2_O_2_ production in H-G treated cells, and this effect was dose-dependent over the tested ATP concentrations (10, 50, 100, and 200 µM; Figs. 6B, S7B). However, the addition of ATP failed to rescue cells from the effects of suramin or PPADS (Figs. 6B, S7B). The results implied that eATP signaling was mediated by PM purinoceptors, and this contributed to the rapid H_2_O_2_ burst triggered by NaCl stress.

#### Cytosolic Ca^2+^

**Figure 7:**
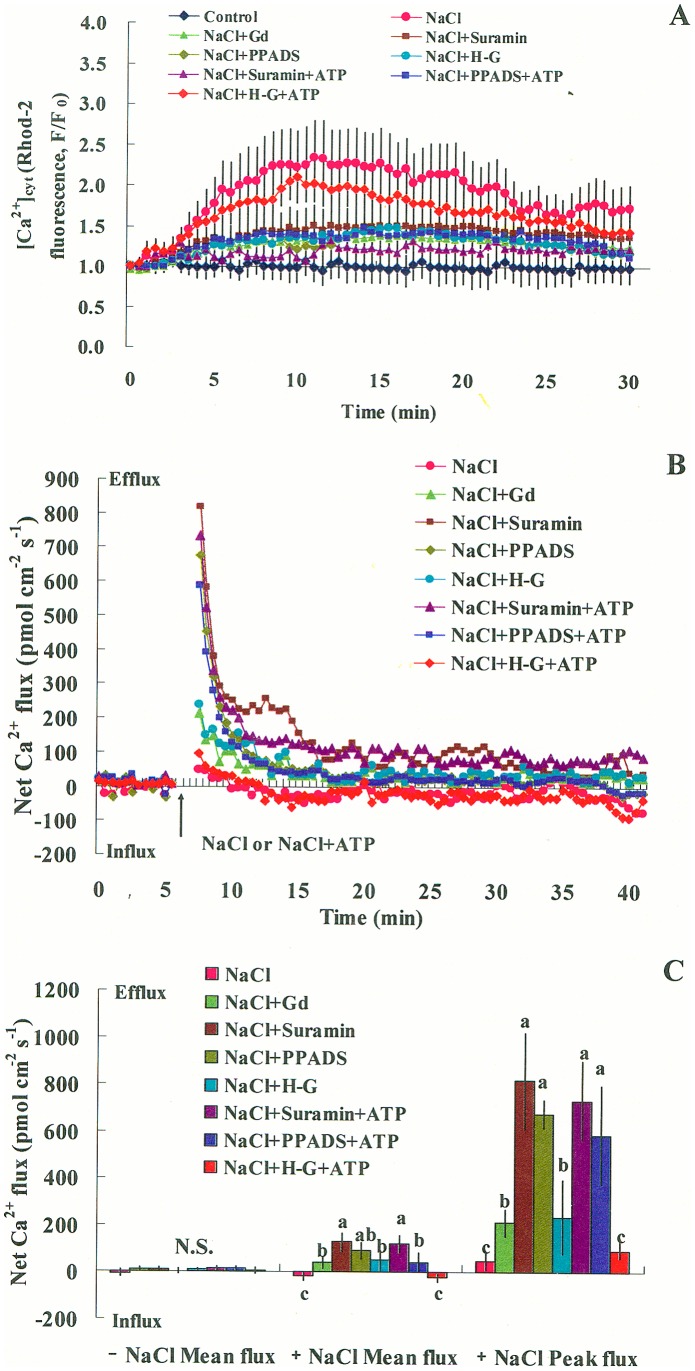
Effects of pharmacological agents on NaCl stress-induced [Ca^2+^]_cyt_ and Ca^2+^ flux in *P. euphratica* cells. Suspended cells were untreated or treated with NaCl (200 mM) or NaCl plus ATP (200 µM) in the presence or absence of suramin (300 µM), PPADS (300 µM), the H-G system (50 mM glucose and 100 units/mL hexokinase), or GdCl_3_ (500 µM). (A) Transient [Ca^2+^]_cyt_. Rhod-2/AM fluorescence intensity was measured in the cytoplasm before (F_0_) and after (F) the treatments. Each point represents the mean of 12 to 15 individual cells from four independent experiments. (B) Transient Ca^2+^ fluxes. Symbols are representative of five to six independent experiments. (C) Peak and mean flux rates of Ca^2+^ before (-) and after (+) the addition of NaCl or NaCl plus ATP. Bars represent the mean of five to six individual cells, and whiskers represent the standard error of the mean. Different letters (a, b, c) denote significant differences between treatments (*P*<0.05). N.S. = no significant difference.

We used a Ca^2+^-sensitive fluorescent dye, Rhod-2/AM, to monitor cytosolic Ca^2+^ in control and stressed cells [41]. Fluorescence detection showed that high NaCl exposure caused an increase in fluorescent intensity that peaked within 10 to 12 min (Fig. 7A). However, the fluorescence response to salt stress could be suppressed by pretreatment with GdCl_3_ (500 µM [35]), suramin, PPADS, or H-G (Fig. 7A). The data revealed that the elevation of [Ca^2+^]_cyt_ in *P. euphratica* cells was dependent on the presence of eATP at the beginning of salt stress. Again, addition of ATP (200 µM) rescued cells from H-G inhibition of salt-induced [Ca^2+^]_cyt_, but not from the effects of suramin or PPADS (Fig. 7A).

To determine whether the salt-elicited [Ca^2+^]_cyt_ resulted from Ca^2+^ entry, we measured the salt-induced Ca^2+^ flux. We observed Ca^2+^ influx after a few minutes of NaCl shock (200 mM), but the flux rate fluctuated over the recording period (Fig. 7B). The Ca^2+^ influx elicited by 200 mM NaCl was not as pronounced as that induced by 100 mM NaCl (Fig. S9) [4]. This was due to the large amount of Ca^2+^ released from the cell walls in the presence of high Na^+^ (200 mM) during SIET recording period (i.e., Na^+^/Ca^2+^ exchange [48]). After exposure to the NaCl shock, cells pretreated with GdCl_3_, suramin, PPADS, or H-G exhibited a dramatic Ca^2+^ efflux (Fig. 7B, C). The flux peaks in these cells were several-fold higher than that elicited by NaCl shock in the absence of inhibitors (Fig. 7B, C). These results showed that the NaCl-induced Ca^2+^ influx in *P. euphratica* cells was blocked by GdCl_3_, suramin, PPADS, or H–G. Addition of ATP (200 µM) was able to rescue the Ca^2+^ influx elicited by NaCl in H–G treated cells, but not in suramin or PPADS-treated cells (Fig. 7B, C).

#### Transient H^+^ fluxes

**Figure 8:**
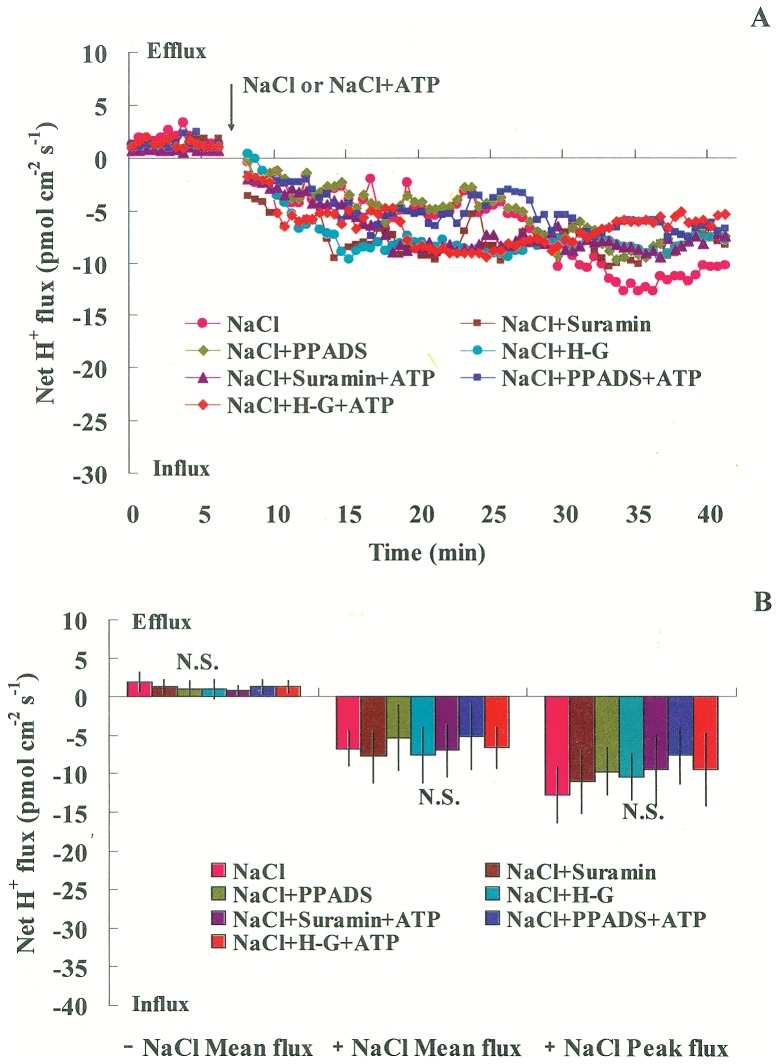
Effects of pharmacological agents on NaCl stress-induced H^+^ flux across the plasma membrane. *P. euphratica* cells were untreated (control) or treated with NaCl (200 mM) or NaCl plus ATP (200 µM) in the presence or absence of suramin (300 µM), PPADS (300 µM), or the H–G system (50 mM glucose and 100 units/mL hexokinase). (A) Transient H^+^ flux. SIET data are representative of six independent experiments. (B) Peak and mean values of H^+^ fluxes before (–) and after (+) the addition of NaCl or NaCl plus ATP. Bars represent the mean of six individual cells, and whiskers represent the standard error of the mean. N.S. = no significant difference.

NaCl-induced alterations in the H^+^ flux have been proposed to serve as a signaling component in sensing ionic stress in *P. euphratica* cells [4]. We investigated whether the salt-induced H^+^ flux was involved in eATP signaling in *P. euphratica* cells. NaCl shock induced a rapid, continuous H^+^ influx across the PM (Fig. 8); this was consistent with our previous report [4]. The pattern of H^+^ flux in NaCl-treated cells was not significantly changed by suramin, PPADS, or H-G, either in the presence or absence of ATP (Fig. 8). These results indicated that the salt-induced H^+^ flux may serve as a signaling component for sensing the ionic effects, rather than the osmotic effects, caused by NaCl stress in *P. euphratica* cells.

## Discussion

### eATP Contributes to Salinity Tolerance of *P. euphratica* Cells

eATP is implicated in the plant response to biotic [20], [21] and abiotic stress [37]. In this study, we found that eATP played a regulatory role in salinity tolerance of *P. euphratica* cells. When eATP signaling was blocked with the H-G trap system or P2 receptor antagonists (suramin and PPADS), *P. euphratica* cells were unable to perform processes of acclimation to the salt medium, including cytosolic Na^+^ exclusion, vacuolar salt compartmentation, K^+^ homeostasis, ROS control, antioxidant defense, and induction of salt-resistant gene expression (Figs. 1, 2, 3, 4, 5, 6, and S3). Moreover, exogenously applied ATP was able to rescue these salt acclimation processes from the effects of H-G, but not from the effects of suramin or PPADS. This suggested that additional ATP was unable to rescue cells when the ATP binding site to the P2 receptor was blocked. In contrast, because the H-G system functioned to deplete ATP, exogenous ATP was able to bind to the hypothetical ATP binding site and rescue the disrupted signal.

We showed that NaCl shock elicited a significant rise in ATP in the ECM (Fig. 1). This finding was consistent with previous reports that showed eATP significantly increased upon hyperosmotic treatment [29], [37]. We noticed that eATP levels returned to basal levels after 20 min of salt treatment (Fig. 1). This was presumably the result of ATP hydrolysis by apyrase, an extracellular nucleotide phosphohydrolases [20], [21]. Maintaining a low eATP level in the ECM is critical for *P. euphratica* cells to cope with high saline environments, because long-term, sustained eATP causes programmed cell death in this salt-resistant species [41].

### eATP Mediates K^+^/Na^+^ Homeostasis in NaCl-stressed Cells

Our results showed that salt-induced increase in eATP contributed to regulating Na^+^ and K^+^ levels in *P. euphratica* cell cultures. *P. euphratica* sustained low cytosolic Na^+^ after 24 h of salt treatment (Fig. 3). This result was consistent with our previous findings that root and callus cells of *P. euphratica* exhibited a strong capacity for excluding Na^+^ via the PM Na^+^/H^+^ antiport system in response to high NaCl exposure [4], [12], [49]. Of note, *P. euphratica* cells accumulated more Na^+^ in the vacuole than in the cytoplasm under salt stress (Fig. 3). This agrees with results from Silva *et al*. (2010), who found that salinized *P. euphratica* suspension cultures displayed high tonoplast Na^+^/H^+^ exchange activity [38]. However, the capacity for cytosolic Na^+^ exclusion and vacuolar ion compartmentation were both diminished by H-G, PPADS, or suramin in salinized cells (Fig. 3). Addition of ATP could rescue the H-G-triggered inhibition of Na^+^ efflux and vacuolar compartmentaion (Fig. 3). These results suggested that salt-induced eATP was implicated in mediating Na^+^/H^+^ antiport across the plasma and vacuolar membranes. Furthermore, qRT-PCR data showed that suramin, PPADS, and H-G could inhibit the salt-induced upregulation of gene expression for the PM Na^+^/H^+^ antiporter (*SOS1*) and PM H^+^-ATPase (*AHA*) in *P. euphratica* cells (Fig. 5). We concluded that the reduced Na^+^ extrusion in inhibitor-treated cells was correlated with the abundance of mRNAs that encode the Na^+^/H^+^ antiport system under salinity stress. When eATP signaling was blocked by suramin, PPADS, or H-G in salinized cells, the salt-induced transcription upregulation of *AVP*, *NHX1*, *VHA-a, VHA-b*, and *VHA-c* was inhibited. This suggested that vacuolar proton pumps (V-H^+^-pyrophosphatase and V-H^+^-ATPase) could not generate H^+^ gradients across the vacuolar membrane, and this led to insufficient Na^+^ compartmentation in the vacuole (Fig. 3). In addition, it was shown that both ATP and H_2_O_2_ are important signaling molecules controlling activity of slow vacuolar (SV) channels [50]. Given the fact that SV channels are Na^+^ permeable and thus directly contribute to Na^+^ sequestration in vacuoles (by preventing its back leak into cytosol), further investigations are necessary to elucidate how salt-induced signaling molecules mediate SV channels and Na^+^ compartmentation. Our previous studies showed that increases in eATP caused increases in the intracellular ATP level [41]. It is highly possible that the increased intracellular ATP enhanced H^+^-coupled transporters (H^+^-ATPase) or regulated other signaling pathways in these cells. However, our experiments did not differentiate between effects due to intracellular ATP and those due to eATP.

NaCl caused membrane depolarization and a net K^+^ efflux in *P. euphratica* cells (Fig. 4). It has repeatedly been shown that salt-induced K^+^ loss was mediated by depolarization-activated K^+^ channels, and this channel-mediated K^+^ flux depended both on MP and H^+^-pumps [4], [19], [51]. In the present study, three pharmacological agents, PPADS, H-G, and suramin, accelerated the salt-induced PM depolarization and K^+^ efflux (Fig. 4). This implied that the PM H^+^-pumps were unable to maintain membrane potentials when eATP was depleted by H-G or when the eATP signaling cascade was blocked by suramin and PPADS. Consistent with this implication, we found that NaCl-induced transcription of the PM H^+^-ATPase was inhibited by suramin, PPADS, or H-G (Fig. 5). We also found that the intracellular Na^+^ distribution and K^+^ fluxes were not affected by these pharmacological agents during the early period of NaCl stress (within 1 h; Figs. 4, S8). This implied that the salt-induced eATP regulated the expression of K^+^/Na^+^ homeostasis genes after a prolonged period of salt stress, rather than exerting a direct effect on protein activity at the initiation of salt treatment.

Interestingly, eATP contributed to the induction of the poplar synaptotagmin gene (*SYT*) during NaCl stress (Fig. 5). In plants, synaptotagmin plays a particularly important role in repairing injured PM under high salt or freezing conditions, and this process is dependent on cytosolic Ca^2+^ signaling [52], [53]. Our data suggested that salt-induced eATP may contribute to PM repair via synaptotagmin-mediated vesicle recycling. However, the underlying mechanism for this process requires further investigation.

### eATP Signaling is Mediated by H_2_O_2_ and [Ca^2+^]_cyt_ in Salinized Cells

In the present study, the results from pharmacological experiments implicated H_2_O_2_ and cytosolic Ca^2+^ involvement in eATP mediation of ionic homeostasis in salt-stressed *P. euphratica* cells (Figs. 6, 7). Much evidence from previous studies has shown that H_2_O_2_ and Ca^2+^ were responsible for the maintenance of cellular K^+^/Na^+^ homeostasis under high saline conditions [1], [2], [4], [8], [9], [14], [15]. In *P. euphratica* cells, the PM Na^+^/H^+^ antiport system was up-regulated by changes in H_2_O_2_ and [Ca^2+^]_cyt_ that were triggered by NaCl shock [4]. In the present study, early changes in H_2_O_2_ and [Ca^2+^]_cyt_ in response to high NaCl were inhibited by the P2 receptor antagonists and the H-G system (Figs. 6, 7). This suggested that the second messengers, Ca^2+^ and ROS, were involved in the eATP-mediated plant response to salt stress [31], [33], [54]. Interestingly, application of ATP reduced the inhibitory effects of the H-G system on salt-induced H_2_O_2_ production and [Ca^2+^]_cyt_ within 1 h of treatment (Figs. 6, 7). Moreover, ATP rescued the effects of H-G treatment on Na^+^ extrusion and K^+^ flux after 24 h of salt treatment (Figs. 3, 4). Therefore, the eATP effects on K^+^/Na^+^ homeostasis in salinized *P. euphratica* were most likely mediated through H_2_O_2_- and Ca^2+^-dependent pathways.

In Arabidopsis, rice, and poplar, high salt treatment stimulated a SOS pathway that caused an increase in Na^+^ extrusion [6]–[8]. It remains unclear whether eATP could mediate salt tolerance independent of SOS3-SOS2-SOS1 signaling. Future studies in Arabidopsis *sos* mutants may facilitate clarification of this issue. In addition to the Ca^2+^-SOS3-SOS2 cascade, a novel signaling component, phosphatidic acid (PA), was shown to be involved in Na^+^ detoxification in Arabidopsis. NaCl stress stimulated PA production and MPK6 activity, which phosphorylated the C-terminal of SOS1 [55]. Interestingly, PA and MAPK have also been reported as intermediates in eATP stimulation of tomato (*Solanum lycopersicum*) and Arabidopsis suspensions [29], [54]. Taken together, these results suggested that eATP initiated different signaling pathways that mediated Na^+^ homeostasis in NaCl-stressed *P. euphratica* cells.

In this study, evidence from the pharmacological experiments suggested that eATP contributed to ROS homeostasis and antioxidant defense in salt stressed *P. euphratica* cells (Figs. 6, S3). In the presence of suramin, PPADS, or H-G, the activity of antioxidant enzymes was inhibited, and H_2_O_2_ production reached high levels after 24-h of NaCl treatment (Figs. 6, S3). This was presumably due to down-regulation of ROS-dependent MAPK cascades, because salt-induced *MPK* expression was inhibited by suramin, PPADS, or H-G in *P. euphratica* cells (Fig. 5). This finding was consistent with previous studies, where eATP was shown to rapidly elevate the mRNA of several MAPK members in Arabidopsis cell suspensions [29]. However, the eATP-induced increase of *MPK3* transcription was not observed in the roots of an Arabidopsis *rhd2/AtrbohC* mutant; this suggested the involvement of ROS in this eATP-related pathway [35]. In addition, MAPK was involved in abscisic acid-induced antioxidant defense, and it acted downstream of ROS production in maize leaves [56]. Therefore, the MAPK cascade was not activated in the absence of early H_2_O_2_ production triggered by eATP (see below); this led to uncontrolled oxidation and cell death in salt stressed *P. euphratica* cells (Figs. 6, S3).

**Figure 9:**
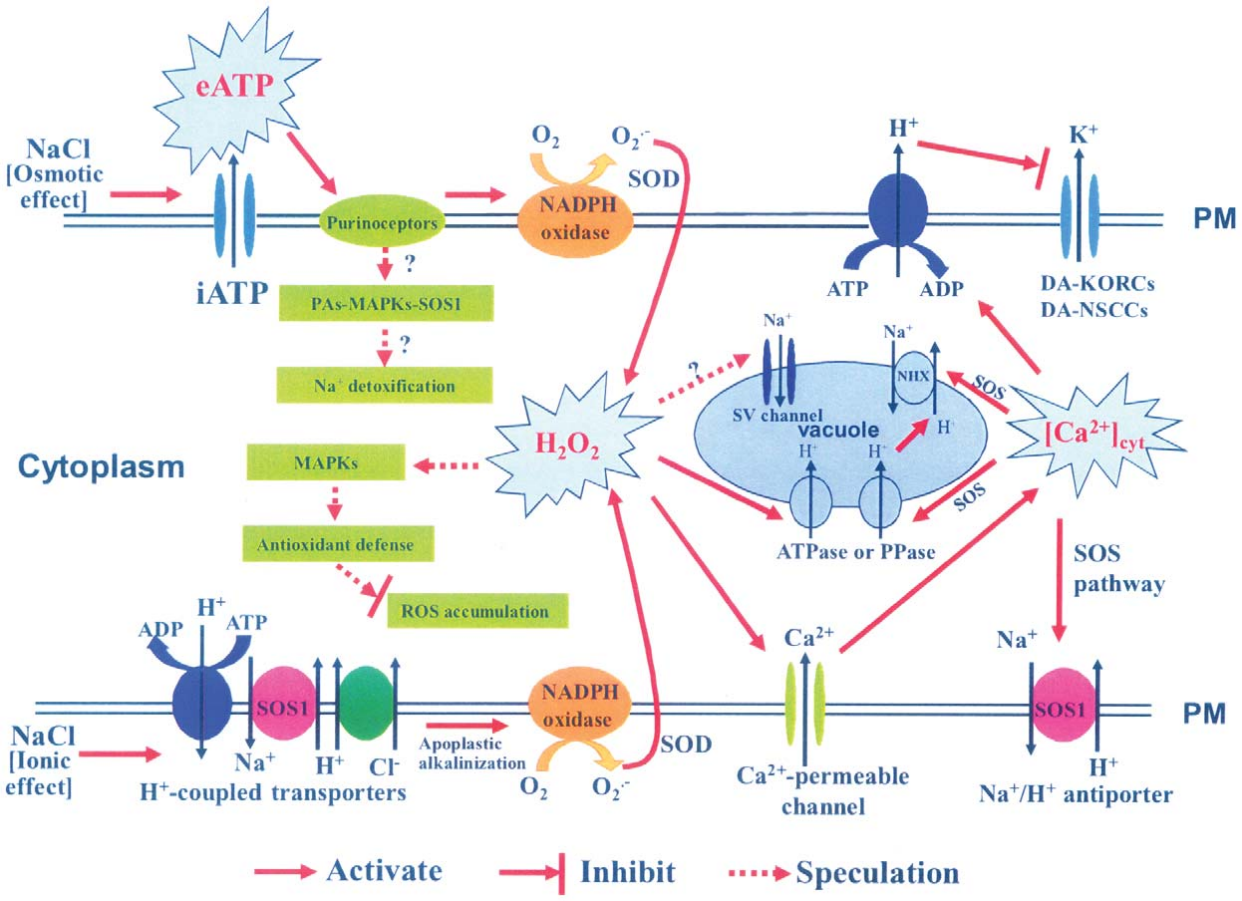
Schematic model shows proposed eATP signals that mediate the NaCl stress response in *P. euphratica* cells. The top line (PM = double line) indicates the molecules involved in the osmotic sensor and associated responses to salt stress. The bottom line (PM = double line) indicates the molecules involved in the ionic sensor and associated responses to salt stress (see text for details). These sensors are separated in this diagram for clarity, but they are not expected to be relegated to separate compartments on the cell membrane.

Previous studies suggested that eATP might cause ROS production through activation of the PM NADPH oxidase [31]. ROS, in turn, could activate Ca^2+^ influx channels, which caused subsequent [Ca^2+^]_cyt_ elevation [31], [35]. In the present study, eATP rapidly increased after *P. euphratica* cells were exposed to an osmotic shock caused by high NaCl (Fig. 1). eATP appeared to activate a receptor in the PM and triggered downstream signaling events; e.g., ROS production and establishment of a Ca^2+^ gradient in the cytosol [29], [33], [57]. Our previous study showed that an ion-specific effect of NaCl was sensed by the PM H^+^-coupled ion transporters (H^+^-ATPase, Na^+^/H^+^ antiporter, and Cl^−/^2H^+^ symporter), which triggered H^+^ influx across the PM, and this led to rises in H_2_O_2_ and [Ca^2+^]_cyt_ in *P. euphratica* cells [4]. In the present study, the pharmacological experiments showed that eATP did not significantly change the pattern of H^+^ flux in NaCl-treated cells (Fig. 8); this suggested that the salt-induced H^+^ flux may serve as an ionic sensor rather than an osmotic sensor. Given these results, we concluded that the H_2_O_2_ and Ca^2+^ signaling in response to high NaCl could be triggered by two sensors: the eATP-activated PM purinergic receptors (osmotic sensing effect) and the PM H^+^-coupled ion transporters (specific salt sensing effect) in the salt-resistant species *P. euphratica* (Fig. 9).

Based on our results, we propose an eATP-regulated stress signaling pathway that confers salinity tolerance in *P. euphratica* cells (Fig. 9). In this pathway, NaCl stress induces a transient increase in [eATP]. This stimulates PM purinergic receptors, which cause the rapid production of H_2_O_2_ through activation of PM NADPH oxidase [35]. This early H_2_O_2_ burst causes the elevation of cytosolic free Ca^2+^, due to influx from PM Ca^2+^ permeable channels. Then, H_2_O_2_, [Ca^2+^]_cyt_ and other feasible signaling components, e.g. PAs, mediate intracellular K^+^/Na^+^ and ROS homeostasis, and a multiple transduction network repairs the plasma membrane that is injured by osmotic shock of high NaCl.

## Supporting Information

Figure S1 **Cell viability, H_2_O_2_, and Ca^2+^ flux in** ***P. euphratica*** **cells in the presence and absence of hormones.** *P. euphratica* cells were incubated in LMS supplemented with or without 0.25 mg L^−1^ benzyladenine (BA) and 0.50 mg L^−1^ α-naphthaleneacetic acid (NAA) for 24 h, then cell viability, H_2_O_2_, and Ca^2+^ flux were measured. Bars represent the means from four independent experiments and whiskers represent the error of the mean. The same letter denotes no significant difference between treatments.
(DOC)

Figure S2 **Effects of H-G on cell viability, H_2_O_2_, and Ca^2+^ flux in** ***P. euphratica*** **cells.** *P. euphratica* cells were incubated in LMS containing an ATP trap (H-G system, 50 mM glucose and 100 units/mL hexokinase) for 6 h, then cell viability, H_2_O_2_, and Ca^2+^ flux were measured. Bars represent the means from four independent experiments and whiskers represent the error of the mean. The same letter denotes no significant difference between treatments.
(DOC)

Figure S3 **Effects of pharmacological agents and ATP on antioxidant enzyme activity in control and NaCl-stressed cells of** ***P. euphratica*** **.** *P. euphratica* cells were treated with 200 mM NaCl for 24 h in the absence and presence of suramin (300 µM), PPADS (300 µM), or H-G (50 mM glucose and 100 units/mL hexokinase). Control cells were incubated in LMS supplemented with or without ATP (200 and 500 µM) for 24 h. Then, the activities of antioxidant enzymes, ascorbic peroxidase (APX), catalase (CAT), and glutathione reductase (GR) were measured; activities are expressed as the amount of ascorbate (ASA), H_2_O_2_, and NADPH consumed, respectively. Each bar represents the mean of four independent experiments, and whiskers represent the standard error of the mean. Different letters (a, b, c, d) indicate significant differences between treatments (*P*<0.05).
(DOC)

Figure S4 **Effects of pharmacological agents and ATP on the expression of salt-responsive genes in no-salt control cells of** ***P. euphratica*** **.** *P. euphratica* cells were treated without (control) or with suramin (300 µM), PPADS (300 µM), H-G (50 mM glucose and 100 units/mL hexokinase), ATP (100 or 200 µM), or ATP_λ_S (200 µM) for 24 h; then, total RNA was isolated for quantitative Real-Time PCR analysis. Each bar represents the mean of four replicates and whiskers represent the standard error of the mean. Different letters (a, b) indicate significant differences between treatments (*P*<0.05).
(DOC)

Figure S5 **Concentration tests for effects of suramin or PPADS on cell viability and H_2_O_2_ accumulation in** ***P. euphratica*** **cells.** Suspended cells were untreated (control) or treated with suramin or PPADS (10, 30, 50, 100, 200, or 300 µM) for 24 h, then cell viability and H_2_O_2_ levels were measured under a fluorescence microscope. (A) Cell viability. Bars represent the mean of three independent experiments in which at least 300 cells were counted. (B) H_2_O_2_ accumulation. Bars represent the mean H_2_O_2_ levels quantified from 45 to 50 individual cells in three independent experiments. Whiskers represent the standard error of the mean. N.S. = no significant difference.
(DOC)

Figure S6 **Effects of P2 receptor antagonists (suramin and PPADS) on the early H_2_O_2_ burst in** ***P. euphratica*** **cells elicited by NaCl or non-hydrolysable ATP (ATP_λ_S).** Suspended cells were incubated with suramin or PPADS (10, 30, 50, 100, 200, and 300 µM) for 2 h, then subjected to (A) 200 mM NaCl or (B) 200 µM ATP_λ_S for 30 min. H_2_O_2_ levels were measured under a fluorescence microscope. Bars represent the mean H_2_O_2_ levels quantified from 45 to 50 individual cells in three independent experiments. Whiskers represent the standard error of the mean. Different letters (a, b, c, d) indicate significant differences between treatments (*P*<0.05).
(DOC)

Figure S7 **Effects of exogenous ATP on Na^+^ flux and early H_2_O_2_ burst in NaCl-stressed** ***P. euphratica*** **cells in the presence and absence of pharmacological agents.** Suspended cells were untreated (control) or pretreated with suramin (300 µM for 2 h), PPADS (300 µM for 2 h), or the H-G system (50 mM glucose and 100 units/mL hexokinase for 6 h). This was followed by exposure to 200 mM NaCl supplemented without or with ATP (10, 50, 100, or 200 µM). (A) Steady Na^+^ fluxes after 24 h or (B) early H_2_O_2_ production after 30 min of NaCl stress. Bars represent the mean of 14–16 (Na^+^ fluxes) and 40–50 (H_2_O_2_) individual cells; whiskers represent the standard error of the mean. Different letters (a, b, c, d) indicate significant differences between treatments (*P*<0.05).
(DOC)

Figure S8 **Effects of pharmacological agents on Na^+^ compartmentation in NaCl-stressed** ***P. euphratica*** **cells.** *P. euphratica* cells were treated with 200 mM NaCl for 1 h in the absence (control) or presence of suramin (300 µM), PPADS (300 µM), or the H-G system (50 mM glucose and 100 units/mL hexokinase). The Na^+^-specific fluorescent probe, CoroNa-Green/AM, was added to detect Na^+^ levels in the cytoplasm and vacuole. Each measurement was based on at least 100 individual cells. Bars are the mean of three independent experiments. Whiskers represent the standard error of the mean. Different letters (a, b) indicate significant differences between treatments (*P*<0.05).
(DOC)

Figure S9 **Transient Ca^2+^ flux in response to NaCl shock in** ***P. euphratica*** **cells.** (A) *P. euphratica* cells were subjected to 100 and 200 mM NaCl shock, respectively. Each point represents the mean value for six individual cells. (B) Mean flux rates of Ca^2+^ before (-) and after (+) the addition of NaCl. Each bar represents the mean of six individual cells and whiskers represent the standard error of the mean. Different letters (a, b) indicate significant differences (*P*<0.05).
(DOC)

Table S1 **Sequences of gene-specific primers used in quantitative Real time PCR analysis.**
(DOC)
