# Supplementary material for: Extracellular ATP Signaling Is Mediated by H2O2 and Cytosolic Ca2+ in the Salt Response of Populus euphratica Cells
Source: PLoS One. 2012 Dec 28;7(12):e53136. doi: 10.1371/journal.pone.0053136 (PMC3532164; doi:10.1371/journal.pone.0053136)
Supplement: Figure S1 — Cell viability, H2O2, and Ca2+ flux in P. euphratica cells in the presence and absence of hormones. P. euphratica cells were incubated in LMS supplemented with or without 0.25 mg L−1 benzyladenine (BA) and 0.50 mg L−1 α-naphthaleneacetic acid (NAA) for 24 h, then cell viability, H2O2, and Ca2+ flux were measured. Bars represent the means from four independent experiments and whiskers represent the error of the mean. The same letter denotes no significant difference between treatments. (DOC) [file pone.0053136.s001.doc]

**Figure S1. Cell viability, H_2_O_2_, and Ca^2+^ flux in *P. euphratica* cells in the presence and absence of hormones.** *P. euphratica* cells were incubated in LMS supplemented with or without 0.25 mg L^-1^ benzyladenine (BA) and 0.50 mg L^-1^ α-naphthaleneacetic acid (NAA) for 24 h, then cell viability, H_2_O_2_, and Ca^2+^ flux were measured. Bars represent the means from four independent experiments and whiskers represent the error of the mean. The same letter denotes no significant difference between treatments.
