# Supplementary material for: Extracellular ATP Signaling Is Mediated by H2O2 and Cytosolic Ca2+ in the Salt Response of Populus euphratica Cells
Source: PLoS One. 2012 Dec 28;7(12):e53136. doi: 10.1371/journal.pone.0053136 (PMC3532164; doi:10.1371/journal.pone.0053136)
Supplement: Figure S2 — Effects of H-G on cell viability, H2O2, and Ca2+ flux in P. euphratica cells. P. euphratica cells were incubated in LMS containing an ATP trap (H-G system, 50 mM glucose and 100 units/mL hexokinase) for 6 h, then cell viability, H2O2, and Ca2+ flux were measured. Bars represent the means from four independent experiments and whiskers represent the error of the mean. The same letter denotes no significant difference between treatments. (DOC) [file pone.0053136.s002.doc]

**Figure S2. Effects of H-G on cell viability, H_2_O_2_, and Ca^2+^ flux in *P. euphratica* cells.** *P. euphratica* cells were incubated in LMS containing an ATP trap (H-G system, 50 mM glucose and 100 units/mL hexokinase) for 6 h, then cell viability, H_2_O_2_, and Ca^2+^ flux were measured. Bars represent the means from four independent experiments and whiskers represent the error of the mean. The same letter denotes no significant difference between treatments.
