# Supplementary material for: Extracellular ATP Signaling Is Mediated by H2O2 and Cytosolic Ca2+ in the Salt Response of Populus euphratica Cells
Source: PLoS One. 2012 Dec 28;7(12):e53136. doi: 10.1371/journal.pone.0053136 (PMC3532164; doi:10.1371/journal.pone.0053136)
Supplement: Figure S3 — Effects of pharmacological agents and ATP on antioxidant enzyme activity in control and NaCl-stressed cells of P. euphratica . P. euphratica cells were treated with 200 mM NaCl for 24 h in the absence and presence of suramin (300 µM), PPADS (300 µM), or H-G (50 mM glucose and 100 units/mL hexokinase). Control cells were incubated in LMS supplemented with or without ATP (200 and 500 µM) for 24 h. Then, the activities of antioxidant enzymes, ascorbic peroxidase (APX), catalase (CAT), and glutathione reductase (GR) were measured; activities are expressed as the amount of ascorbate (ASA), H2O2, and NADPH consumed, respectively. Each bar represents the mean of four independent experiments, and whiskers represent the standard error of the mean. Different letters (a, b, c, d) indicate significant differences between treatments (P<0.05). (DOC) [file pone.0053136.s003.doc]

**Figure S3. Effects of pharmacological agents and ATP on antioxidant enzyme activity in control and NaCl-stressed cells of *P. euphratica*.** *P. euphratica* cells were treated with 200 mM NaCl for 24 h in the absence and presence of suramin (300 μM), PPADS (300 μM), or H-G (50 mM glucose and 100 units/mL hexokinase). Control cells were incubated in LMS supplemented with or without ATP (200 and 500 μM) for 24 h. Then, the activities of antioxidant enzymes, ascorbic peroxidase (APX), catalase (CAT), and glutathione reductase (GR) were measured; activities are expressed as the amount of ascorbate (ASA), H_2_O_2_, and NADPH consumed, respectively. Each bar represents the mean of four independent experiments, and whiskers represent the standard error of the mean. Different letters (a, b, c, d) indicate significant differences between treatments (*P*< 0.05).
