# Supplementary material for: Extracellular ATP Signaling Is Mediated by H2O2 and Cytosolic Ca2+ in the Salt Response of Populus euphratica Cells
Source: PLoS One. 2012 Dec 28;7(12):e53136. doi: 10.1371/journal.pone.0053136 (PMC3532164; doi:10.1371/journal.pone.0053136)
Supplement: Figure S4 — Effects of pharmacological agents and ATP on the expression of salt-responsive genes in no-salt control cells of P. euphratica . P. euphratica cells were treated without (control) or with suramin (300 µM), PPADS (300 µM), H-G (50 mM glucose and 100 units/mL hexokinase), ATP (100 or 200 µM), or ATPλS (200 µM) for 24 h; then, total RNA was isolated for quantitative Real-Time PCR analysis. Each bar represents the mean of four replicates and whiskers represent the standard error of the mean. Different letters (a, b) indicate significant differences between treatments (P<0.05). (DOC) [file pone.0053136.s004.doc]

**Figure S4. Effects of pharmacological agents and ATP on the expression of salt-responsive genes in no-salt control cells of *P. euphratica*.** *P. euphratica* cells were treated without (control) or with suramin (300 μM), PPADS (300 μM), H-G (50 mM glucose and 100 units/mL hexokinase), ATP (100 or 200 μM), or ATP_λ_S (200 μM) for 24 h; then, total RNA was isolated for quantitative Real-Time PCR analysis. Each bar represents the mean of four replicates and whiskers represent the standard error of the mean. Different letters (a, b) indicate significant differences between treatments (*P* < 0.05).
