# Supplementary material for: Extracellular ATP Signaling Is Mediated by H2O2 and Cytosolic Ca2+ in the Salt Response of Populus euphratica Cells
Source: PLoS One. 2012 Dec 28;7(12):e53136. doi: 10.1371/journal.pone.0053136 (PMC3532164; doi:10.1371/journal.pone.0053136)
Supplement: Figure S5 — Concentration tests for effects of suramin or PPADS on cell viability and H2O2 accumulation in P. euphratica cells. Suspended cells were untreated (control) or treated with suramin or PPADS (10, 30, 50, 100, 200, or 300 µM) for 24 h, then cell viability and H2O2 levels were measured under a fluorescence microscope. (A) Cell viability. Bars represent the mean of three independent experiments in which at least 300 cells were counted. (B) H2O2 accumulation. Bars represent the mean H2O2 levels quantified from 45 to 50 individual cells in three independent experiments. Whiskers represent the standard error of the mean. N.S. = no significant difference. (DOC) [file pone.0053136.s005.doc]

**Figure S5. Concentration tests for effects of suramin or PPADS on cell viability and H_2_O_2_ accumulation in *P. euphratica* cells.** Suspended cells were untreated (control) or treated with suramin or PPADS (10, 30, 50, 100, 200, or 300 μM) for 24 h, then cell viability and H_2_O_2_ levels were measured under a fluorescence microscope. (A) Cell viability. Bars represent the mean of three independent experiments in which at least 300 cells were counted. (B) H_2_O_2_ accumulation. Bars represent the mean H_2_O_2_ levels quantified from 45 to 50 individual cells in three independent experiments. Whiskers represent the standard error of the mean. N.S. = no significant difference.
