# Supplementary material for: Extracellular ATP Signaling Is Mediated by H2O2 and Cytosolic Ca2+ in the Salt Response of Populus euphratica Cells
Source: PLoS One. 2012 Dec 28;7(12):e53136. doi: 10.1371/journal.pone.0053136 (PMC3532164; doi:10.1371/journal.pone.0053136)
Supplement: Figure S6 — Effects of P2 receptor antagonists (suramin and PPADS) on the early H2O2 burst in P. euphratica cells elicited by NaCl or non-hydrolysable ATP (ATPλS). Suspended cells were incubated with suramin or PPADS (10, 30, 50, 100, 200, and 300 µM) for 2 h, then subjected to (A) 200 mM NaCl or (B) 200 µM ATPλS for 30 min. H2O2 levels were measured under a fluorescence microscope. Bars represent the mean H2O2 levels quantified from 45 to 50 individual cells in three independent experiments. Whiskers represent the standard error of the mean. Different letters (a, b, c, d) indicate significant differences between treatments (P<0.05). (DOC) [file pone.0053136.s006.doc]

**A**

**B**

**Figure S6.** **Effects of P2 receptor** [**antagonist**](app:ds:antagonist)**s (suramin and PPADS) on the early H_2_O_2_ burst in *P. euphratica* cells elicited by NaCl or non-hydrolysable ATP (ATP_λ_S).** Suspended cells were incubated with suramin or PPADS (10, 30, 50, 100, 200, and 300 μM) for 2 h, then subjected to (A) 200 mM NaCl or (B) 200 μM ATP_λ_S for 30 min. H_2_O_2_ levels were measured under a fluorescence microscope. Bars represent the mean H_2_O_2_ levels quantified from 45 to 50 individual cells in three independent experiments. Whiskers represent the standard error of the mean. Different letters (a, b, c, d) indicate significant differences between treatments (*P*< 0.05).
