# Supplementary material for: Extracellular ATP Signaling Is Mediated by H2O2 and Cytosolic Ca2+ in the Salt Response of Populus euphratica Cells
Source: PLoS One. 2012 Dec 28;7(12):e53136. doi: 10.1371/journal.pone.0053136 (PMC3532164; doi:10.1371/journal.pone.0053136)
Supplement: Figure S7 — Effects of exogenous ATP on Na+ flux and early H2O2 burst in NaCl-stressed P. euphratica cells in the presence and absence of pharmacological agents. Suspended cells were untreated (control) or pretreated with suramin (300 µM for 2 h), PPADS (300 µM for 2 h), or the H-G system (50 mM glucose and 100 units/mL hexokinase for 6 h). This was followed by exposure to 200 mM NaCl supplemented without or with ATP (10, 50, 100, or 200 µM). (A) Steady Na+ fluxes after 24 h or (B) early H2O2 production after 30 min of NaCl stress. Bars represent the mean of 14–16 (Na+ fluxes) and 40–50 (H2O2) individual cells; whiskers represent the standard error of the mean. Different letters (a, b, c, d) indicate significant differences between treatments (P<0.05). (DOC) [file pone.0053136.s007.doc]

**A**

**B**

**Figure S7. Effects of exogenous ATP on Na^+^ flux and early H_2_O_2_ burst in NaCl-stressed *P. euphratica* cells in the presence and absence of pharmacological agents.** Suspended cells were untreated (control) or pretreated with suramin (300 μM for 2 h), PPADS (300 μM for 2 h), or the H-G system (50 mM glucose and 100 units/mL hexokinase for 6 h). This was followed by exposure to 200 mM NaCl supplemented without or with ATP (10, 50, 100, or 200 µM). (A) Steady Na^+^ fluxes after 24 h or (B) early H_2_O_2_ production after 30 min of NaCl stress. Bars represent the mean of 14-16 (Na^+^ fluxes) and 40-50 (H_2_O_2_) individual cells; whiskers represent the standard error of the mean. Different letters (a, b, c, d) indicate significant differences between treatments (*P* < 0.05).
