# Supplementary material for: Extracellular ATP Signaling Is Mediated by H2O2 and Cytosolic Ca2+ in the Salt Response of Populus euphratica Cells
Source: PLoS One. 2012 Dec 28;7(12):e53136. doi: 10.1371/journal.pone.0053136 (PMC3532164; doi:10.1371/journal.pone.0053136)
Supplement: Figure S8 — Effects of pharmacological agents on Na+ compartmentation in NaCl-stressed P. euphratica cells. P. euphratica cells were treated with 200 mM NaCl for 1 h in the absence (control) or presence of suramin (300 µM), PPADS (300 µM), or the H-G system (50 mM glucose and 100 units/mL hexokinase). The Na+-specific fluorescent probe, CoroNa-Green/AM, was added to detect Na+ levels in the cytoplasm and vacuole. Each measurement was based on at least 100 individual cells. Bars are the mean of three independent experiments. Whiskers represent the standard error of the mean. Different letters (a, b) indicate significant differences between treatments (P<0.05). (DOC) [file pone.0053136.s008.doc]

**Figure S8. Effects of pharmacological agents on Na^+^ compartmentation in NaCl-stressed *P. euphratica* cells.** *P. euphratica* cells were treated with 200 mM NaCl for 1 h in the absence (control) or presence of suramin (300 μM), PPADS (300 μM), or the H-G system (50 mM glucose and 100 units/mL hexokinase). The Na^+^-specific fluorescent probe, CoroNa-Green/AM, was added to detect Na^+^ levels in the cytoplasm and vacuole. Each measurement was based on at least 100 individual cells. Bars are the mean of three independent experiments. Whiskers represent the standard error of the mean. Different letters (a, b) indicate significant differences between treatments (*P* < 0.05).
