# Supplementary material for: Extracellular ATP Signaling Is Mediated by H2O2 and Cytosolic Ca2+ in the Salt Response of Populus euphratica Cells
Source: PLoS One. 2012 Dec 28;7(12):e53136. doi: 10.1371/journal.pone.0053136 (PMC3532164; doi:10.1371/journal.pone.0053136)
Supplement: Figure S9 — Transient Ca2+ flux in response to NaCl shock in P. euphratica cells. (A) P. euphratica cells were subjected to 100 and 200 mM NaCl shock, respectively. Each point represents the mean value for six individual cells. (B) Mean flux rates of Ca2+ before (-) and after (+) the addition of NaCl. Each bar represents the mean of six individual cells and whiskers represent the standard error of the mean. Different letters (a, b) indicate significant differences (P<0.05). (DOC) [file pone.0053136.s009.doc]

**A**

**B**

**Figure S9. Transient Ca^2+^ flux in response to NaCl shock in *P. euphratica* cells.** (A) *P. euphratica* cells were subjected to 100 and 200 mM NaCl shock, respectively. Each point represents the mean value for six individual cells. (B) Mean flux rates of Ca^2+^ before (-) and after (+) the addition of NaCl. Each bar represents the mean of six individual cells and whiskers represent the standard error of the mean. Different letters (a, b) indicate significant differences (*P*< 0.05).
