# Supplementary material for: Extracellular ATP Signaling Is Mediated by H2O2 and Cytosolic Ca2+ in the Salt Response of Populus euphratica Cells
Source: PLoS One. 2012 Dec 28;7(12):e53136. doi: 10.1371/journal.pone.0053136 (PMC3532164; doi:10.1371/journal.pone.0053136)
Supplement: Table S1 — Sequences of gene-specific primers used in quantitative Real time PCR analysis. (DOC) [file pone.0053136.s010.doc]

**Table S1. Sequences of gene-specific primers used in quantitative** Real time PCR analysis

| **Gene annotation/description** | **Gene abbreviation** | **Forward primer**  **5´— 3´** | **Reverse primer**  **5´— 3´** | **GenBank accession numbers** |
| --- | --- | --- | --- | --- |
| Plasma membrane H^+^ -ATPase | *AHA* | TCAGATAAGTTTGGTGTCAGGTC | CGGATTGCGAATTTGAGGATA | XM_002330768.1 |
| Vacuolar H^+^-pyrophosphatase | *AVP* | TAACTGGATATGGACTTGGAGGA | TCAGCGATCACAGCAGGATT | XM_002331026.1 |
| Vacuolar Na^+^/H^+^ exchanger | *NHX1* | GATGAATTTCGGTTTGAGGATG | ACCAATGGCAAGGGCAGTA | FJ589739.1 |
| Vacuolar H^+^-ATPase subunit a | *VHA-a* | TTATCAAACATCGGTTGGGAG | TAGTTCAAGTGCTGTTGGTCTCA | AJ775942.1 |
| Vacuolar H^+^-ATPase subunit b | *VHA-b* | GATGATTCAGACAGGGATTTCG | GTCAGACTTCTCCAATCGCTTTAC | CV240326.1 |
| Vacuolar H^+^-ATPase subunit c | *VHA-c* | AATCATCTTCTCCGATCTCGTC | CCCAAAACTCCAGCCATAACA | DT495947.1 |
| Plasma membrane Na^+^/H^+^ antiporter | *SOS1* | CATTTGTGCTGCATTTCTACG | TTCCTTTTCTTCCAATAACCCAAC | DQ517530.1 |
| Synaptotagmin | *SYT* | GAAGTCATCAGCACCTCGTCT | GGTCTCCACTGCAACTCTATTT | XM_002306146.1 |
| Mitogen-activated protein kinase | *MPK* | GCGATAGATGTGTGGTCTGTAGGT | AGCGGTTGAAAAAGTGGGAA | XM_002298414.1 |
